# Serum Neurofilament Light Chain in Diabetic Polyneuropathy: Associations with Neuropathy Burden, Balance Impairment, and Fear of Falling

**DOI:** 10.3390/jcm15187142

**Published:** 2026-09-15

**Authors:** Zeynep Karakuzu Güngör, Mürvet Algemi, Hanişe Ozkan Sonay, Murat Akarsu

**Affiliations:** 1 Department of Physical Medicine and Rehabilitation, Kanuni Sultan Süleyman Training and Research Hospital, Istanbul 34303, Turkey; 2 Department of Clinical Biochemistry, Kanuni Sultan Süleyman Training and Research Hospital, Istanbul 34303, Turkey; 3 Department of Internal Medicine, Kanuni Sultan Süleyman Training and Research Hospital, Istanbul 34303, Turkey

**Keywords:** diabetic polyneuropathy, neurofilament light chain, balance, fear of falling, falls, biomarker

## Abstract

**Objective**: To compare serum neurofilament light chain (NfL) concentrations between individuals with type 2 diabetes mellitus (T2DM) with and without diabetic polyneuropathy (DPN), and to examine associations with neuropathy burden, balance impairment, fear of falling, and fall history. **Methods**: This analytical cross-sectional study included 88 participants with T2DM (44 with DPN and 44 without DPN). Serum NfL was measured by enzyme-linked immunosorbent assay (ELISA); neuropathy burden was assessed with the Michigan Neuropathy Screening Instrument (MNSI), balance with the Berg Balance Scale (BBS), and fear of falling with the Falls Efficacy Scale–International (FES-I). **Results**: Serum NfL was higher in DPN than non-DPN participants [3.47 (2.68–4.77) vs. 2.47 (1.48–3.07) pg/mL; *p* < 0.001]. NfL correlated with MNSI-A (ρ = 0.562), MNSI-B (ρ = 0.550), FES-I (ρ = 0.431), falls (ρ = 0.344), and BBS (ρ = −0.450) (all *p* ≤ 0.001). In the comprehensively adjusted model, the NfL association was attenuated (adjusted odds ratio [aOR] = 1.352; 95% confidence interval [CI], 0.991–1.844; *p* = 0.057). NfL showed moderate discrimination (area under the receiver operating characteristic curve [AUC] = 0.716; 95% CI, 0.607–0.825); the internally derived Youden threshold was ≥2.823 pg/mL (sensitivity 72.7%, specificity 65.9%). **Conclusions**: NfL may be a complementary marker of DPN-related neuropathy and functional burden, but the adjusted association was attenuated and the study-specific threshold requires external validation. *Study Registration:* ClinicalTrials.gov identifier NCT07480330. The study was prospectively registered; the initial registry release occurred on 14 March 2026, before study initiation on 25 March 2026.

## 1. Introduction

Diabetes mellitus (DM) is a chronic metabolic disease that can lead to numerous microvascular and macrovascular complications. Diabetic polyneuropathy (DPN) is one of the most common complications of diabetes and can substantially impair patients’ functional capacity and quality of life by affecting sensory and motor nerve function [1,2]. The clinical consequences of DPN are not limited to neuropathic symptoms and sensory loss; impaired proprioception, motor dysfunction, and muscle weakness may contribute to impaired postural control, balance deficits, and an increased risk of falls [3,4]. Timar et al. reported poorer balance performance and greater fear of falling in patients with DPN [3], while a recent study demonstrated that neuropathy burden was independently associated with both balance impairment and fear of falling [4].

Although clinical examination and nerve conduction studies play a central role in the assessment of DPN, there is growing interest in circulating biomarkers that may reflect neuroaxonal damage. Neurofilament light chain (NfL) is a structural component of the axonal cytoskeleton and is released into the circulation following neuroaxonal injury. NfL, which can be measured in serum or plasma, has been investigated as a potential biomarker of neuroaxonal injury in various central and peripheral nervous system disorders [5,6].

In recent years, evidence regarding the role of NfL in DPN has been accumulating. Maalmi et al. reported that serum NfL levels were associated with diabetic sensorimotor polyneuropathy and peripheral nerve dysfunction [7]. Määttä et al. also demonstrated an association between serum NfL levels and peripheral nerve damage in patients with type 2 DM [8], and subsequent longitudinal studies provided further evidence supporting the potential role of NfL in early DPN [9]. More recent evidence suggests that serum NfL levels may be associated with structural and functional nerve fiber loss in both painful and painless DPN [10].

The primary objective was to compare serum NfL concentrations between participants with T2DM with and without DPN. Secondary objectives were to examine associations between NfL and neuropathy burden, balance impairment, fear of falling, and fall history. Exploratory objectives were to evaluate the adjusted association between NfL and DPN, the discriminatory performance of NfL, differences across electrophysiological DPN severity categories, and associations within the DPN subgroup.

## 2. Materials and Methods

### 2.1. Study Design and Participants

**Figure 1:**
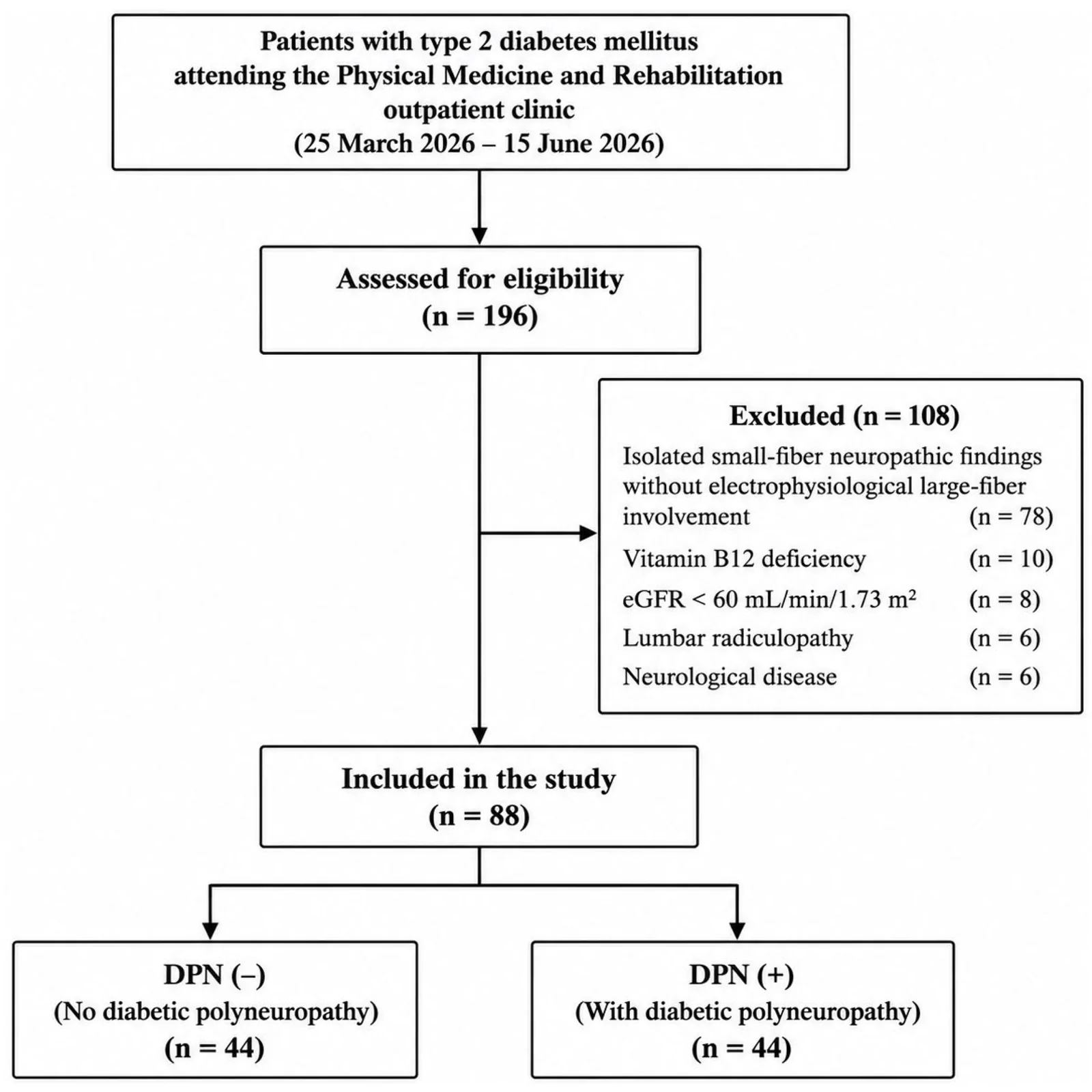
Flow diagram of participant recruitment, exclusions, and group allocation.

This analytical cross-sectional study was conducted among patients with T2DM attending the Physical Medicine and Rehabilitation outpatient clinic between 25 March and 15 June 2026 using a non-probability sampling approach. A total of 196 patients were assessed for eligibility. Of these, 108 were excluded: 78 had isolated small-fiber neuropathic findings without electrophysiological evidence of large-fiber involvement, 10 had vitamin B12 deficiency, 8 had an eGFR < 60 mL/min/1.73 m^2^, 6 had lumbar radiculopathy, and 6 had neurological disease. The final study population comprised 88 participants, including 44 with DPN and 44 without DPN (Figure 1).

Patients with peripheral neuropathy due to causes other than diabetes (e.g., vitamin B12 deficiency, hypothyroidism, alcohol use, or chemotherapy), an estimated glomerular filtration rate (eGFR) < 60 mL/min/1.73 m^2^, active infection or malignancy, inflammatory or autoimmune neurological disease, central nervous system disease, lumbar radiculopathy, peripheral entrapment neuropathy, severe visual or balance impairment that could affect the assessments, pregnancy, or cognitive impairment that precluded completion of the questionnaires were excluded. Patients with isolated small-fiber neuropathy without electrophysiological evidence of large-fiber involvement were also excluded.

A total of 88 patients were included in the study, with 44 patients in the DPN (+) group and 44 in the DPN (−) group.

### 2.2. Clinical Assessment

Demographic and clinical information was obtained for all participants, including age, sex, body mass index (BMI), diabetes duration, smoking status, and other relevant characteristics. Glycated hemoglobin (HbA1c) and estimated glomerular filtration rate (eGFR) values were recorded as laboratory variables.

Neuropathy-related symptoms and examination findings were evaluated with the Michigan Neuropathy Screening Instrument (MNSI) [11]. MNSI-A and MNSI-B were recorded as continuous measures of neuropathy symptoms and examination burden and were not used with a prespecified diagnostic cut-off for DPN group assignment. Falls were recorded retrospectively as participant-reported events during the preceding 12 months and were interpreted as fall history rather than prospective fall risk.

Functional evaluation included assessment of balance with the Berg Balance Scale (BBS) and fear of falling with the Falls Efficacy Scale–International (FES-I). Validated and reliable Turkish versions of both instruments were used [12,13]. Participants were also asked to report the number of falls they had experienced during the preceding 12 months.

### 2.3. Electrophysiological Assessment

Nerve conduction studies were performed and interpreted as part of routine clinical practice in the hospital electrophysiology unit by neurologists who were not involved in the study and were unaware of serum NfL results. DPN status was determined from the overall clinical assessment, MNSI findings, and the conclusion of the formal electrophysiological report. Participants were classified as DPN (+) when the formal nerve conduction study was interpreted as showing findings compatible with polyneuropathy; participants without clinical or electrophysiological evidence of polyneuropathy were classified as DPN (−). Routine studies included the median, ulnar, sural, peroneal, and tibial nerves. No study-specific retrospective algorithm based on a predefined minimum number of abnormal nerves or newly defined nerve-conduction cut-offs was applied. Individual numerical nerve-conduction parameters and reference ranges were not available in a structured form for separate analysis. Complete blinding to clinical information cannot be stated because the examinations were performed as part of routine clinical care.

Among participants with DPN, electrophysiological severity was categorized as mild, moderate, or severe according to the grading documented by the neurologist in the original routine clinical electrophysiological report. No post hoc numerical electrophysiological severity score was constructed.

### 2.4. Serum NfL Measurement

Following an overnight fast, venous blood samples were collected from all participants. Serum was separated by centrifugation and stored at −80 °C until analysis. Serum NfL was measured using the H. Serum NfL was measured using the Human NEFL ELISA Kit (Elabscience Biotechnology Co., Ltd., Wuhan, China; Catalog No. E-EL-H6203) according to the manufacturer’s instructions. Assay sensitivity was 1.03 pg/mL and the stated detection range was 3.13–200 pg/mL. Manufacturer-reported intra-assay CVs ranged from 4.12% to 6.07%, and inter-assay CVs from 6.63% to 8.69%. Samples were measured in duplicate, randomly allocated across the assay plate, and analyzed by laboratory personnel blinded to DPN group allocation. Samples were thawed only once for analysis.

### 2.5. Sample Size Calculation

The sample size was determined a priori based on an expected moderate effect size (Cohen’s d = 0.65) for the difference in serum NfL levels between two independent groups, with a two-sided significance level of 0.05 and 80% statistical power. The analysis indicated that at least 40 participants per group were required. The final study population comprised 88 participants, with 44 individuals in each group.

### 2.6. Ethics Approval and Study Registration

Ethical approval was granted by the Scientific Research Ethics Committee of Kanuni Sultan Süleyman Training and Research Hospital, University of Health Sciences (Decision No. 69; 11 March 2026). The study was conducted in accordance with the Declaration of Helsinki, and written informed consent was obtained from all participants before enrollment. The study was prospectively registered at ClinicalTrials.gov (NCT07480330); the initial registry release occurred on 14 March 2026, before study initiation on 25 March 2026.

### 2.7. Statistical Analysis

Statistical analyses were conducted using SPSS (Statistical Package for the Social Sciences), version 25.0. Categorical data are reported as frequencies and percentages. Continuous data are summarized as mean ± standard deviation when normally distributed and as median with interquartile range when the distribution was non-normal. Distributional assumptions were examined through visual inspection together with the Shapiro–Wilk test.

Two-group comparisons of continuous variables were performed with the independent-samples *t*-test for normally distributed data and the Mann–Whitney U test for non-normally distributed data. Categorical data were examined using the chi-square test or, where appropriate, the Fisher–Freeman–Halton exact test. Comparisons across more than two groups were conducted using one-way analysis of variance (ANOVA) for parametric data and the Kruskal–Wallis test for non-parametric data. Significant Kruskal–Wallis results were followed by pairwise testing with Dunn–Bonferroni adjustment.

Spearman correlation coefficients were calculated for relationships between serum NfL and clinical or functional measures. Full-cohort and DPN-subgroup correlation analyses were exploratory. No formal multiplicity correction was applied to these exploratory correlations; they were interpreted as hypothesis-generating. Dunn–Bonferroni-adjusted post hoc comparisons were used following significant Kruskal–Wallis tests.

Binary logistic regression was performed with DPN status as the dependent variable. The principal multivariable model simultaneously included age, sex, BMI, diabetes duration, HbA1c, eGFR, and serum NfL, selected on the basis of clinical and biological relevance rather than univariable statistical significance. Adjusted odds ratios (aORs) with 95% confidence intervals (CIs) were reported. For NfL, the aOR represents the change in the odds of DPN associated with a 1-pg/mL increase in NfL after adjustment for the included covariates.

ROC analysis was used to quantify the discriminatory performance of serum NfL for DPN. The Youden index identified an exploratory, internally derived threshold. Because the threshold was derived and evaluated in the same dataset, it was not considered a clinically validated diagnostic cut-off.

## 3. Results

**Figure 2:**
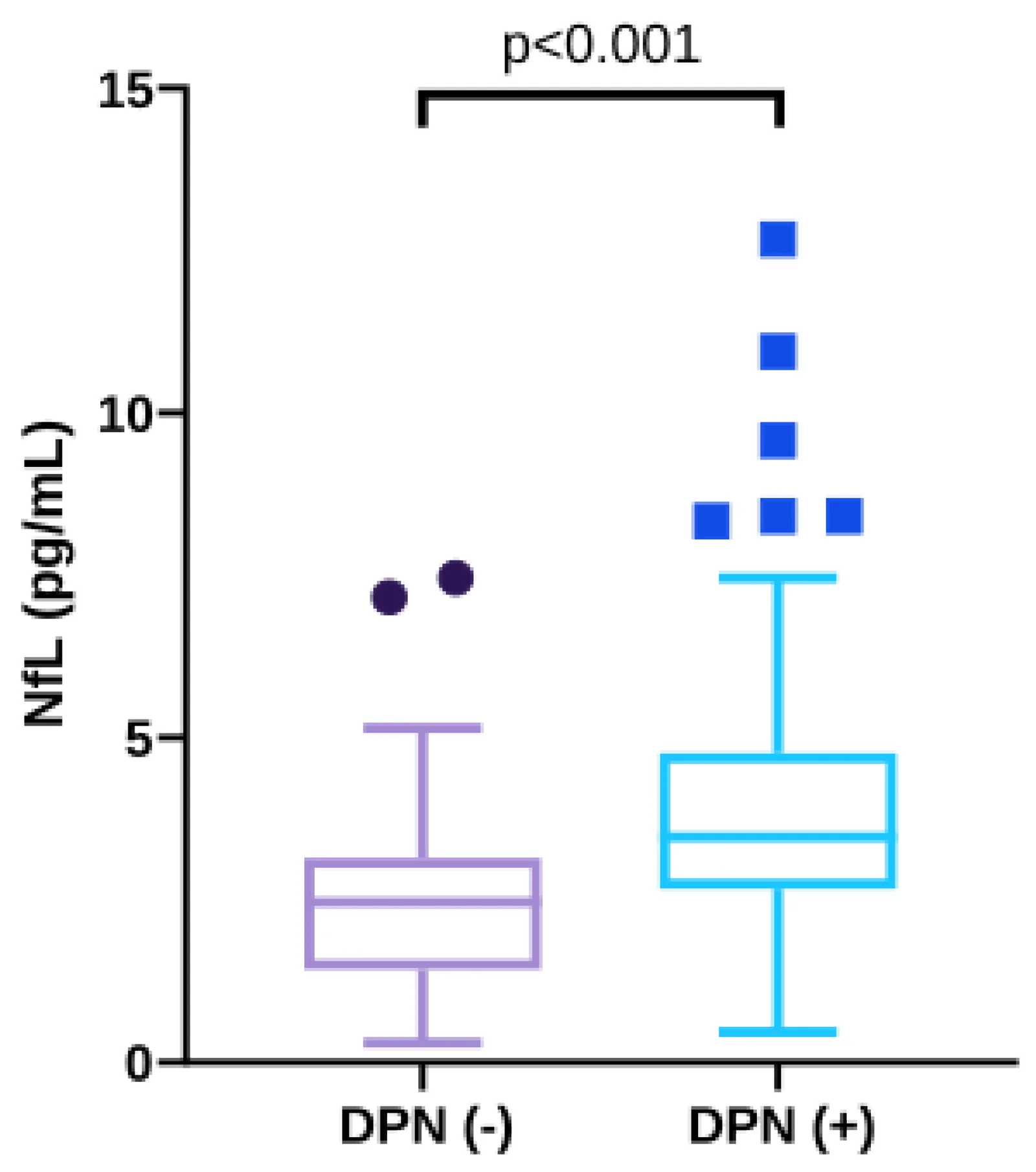
Distribution of serum NfL levels in participants with and without diabetic polyneuropathy.

The study population comprised 88 individuals with T2DM, equally distributed between the DPN (+) and DPN (−) groups (*n* = 44 each). Serum NfL concentrations were significantly higher in participants with DPN than in those without DPN [median (IQR), 3.47 (2.68–4.77) vs. 2.47 (1.48–3.07) pg/mL; *p* < 0.001] (Figure 2). Table 1 summarizes the revised demographic and clinical comparisons.

Within the DPN (+) group, electrophysiological severity was mild in 20 participants, moderate in 19, and severe in 5. MNSI-A and MNSI-B were analyzed as continuous measures of neuropathy burden rather than as between-group diagnostic variables. DPN duration was not included in the analyses because a sufficiently reproducible operational definition of DPN onset was not available.

Exploratory correlations between serum NfL and clinical and functional measures are summarized in Table 2. Higher NfL was associated with greater neuropathy burden, greater fear of falling, poorer balance performance, and a greater number of retrospectively reported falls; an inverse association was observed with BMI.

In the comprehensively adjusted model including age, sex, BMI, diabetes duration, HbA1c, eGFR, and serum NfL, HbA1c remained associated with DPN (aOR = 1.815; 95% CI, 1.158–2.843; *p* = 0.009). The NfL effect estimate remained positive but was attenuated and did not reach conventional statistical significance (aOR = 1.352; 95% CI, 0.991–1.844; *p* = 0.057) (Table 3).

ROC analysis showed moderate discriminatory performance of serum NfL for DPN (AUC = 0.716; 95% CI, 0.607–0.825; *p* < 0.001). At the exploratory, internally derived Youden threshold of ≥2.823 pg/mL, sensitivity was 72.7% and specificity was 65.9% (Table 4).

In exploratory analyses, participants with DPN were classified as mild (*n* = 20), moderate (*n* = 19), or severe (*n* = 5) according to electrophysiological severity. Diabetes duration, MNSI-A, BBS, FES-I, and number of falls differed across severity categories. Given the small and unequal subgroup sizes, these findings should be interpreted cautiously (Table 5).

Serum NfL concentrations did not differ significantly across mild, moderate, and severe electrophysiological DPN categories (*p* = 0.152). This exploratory nonsignificant finding should not be interpreted as evidence of absence of an association.

**Figure 3:**
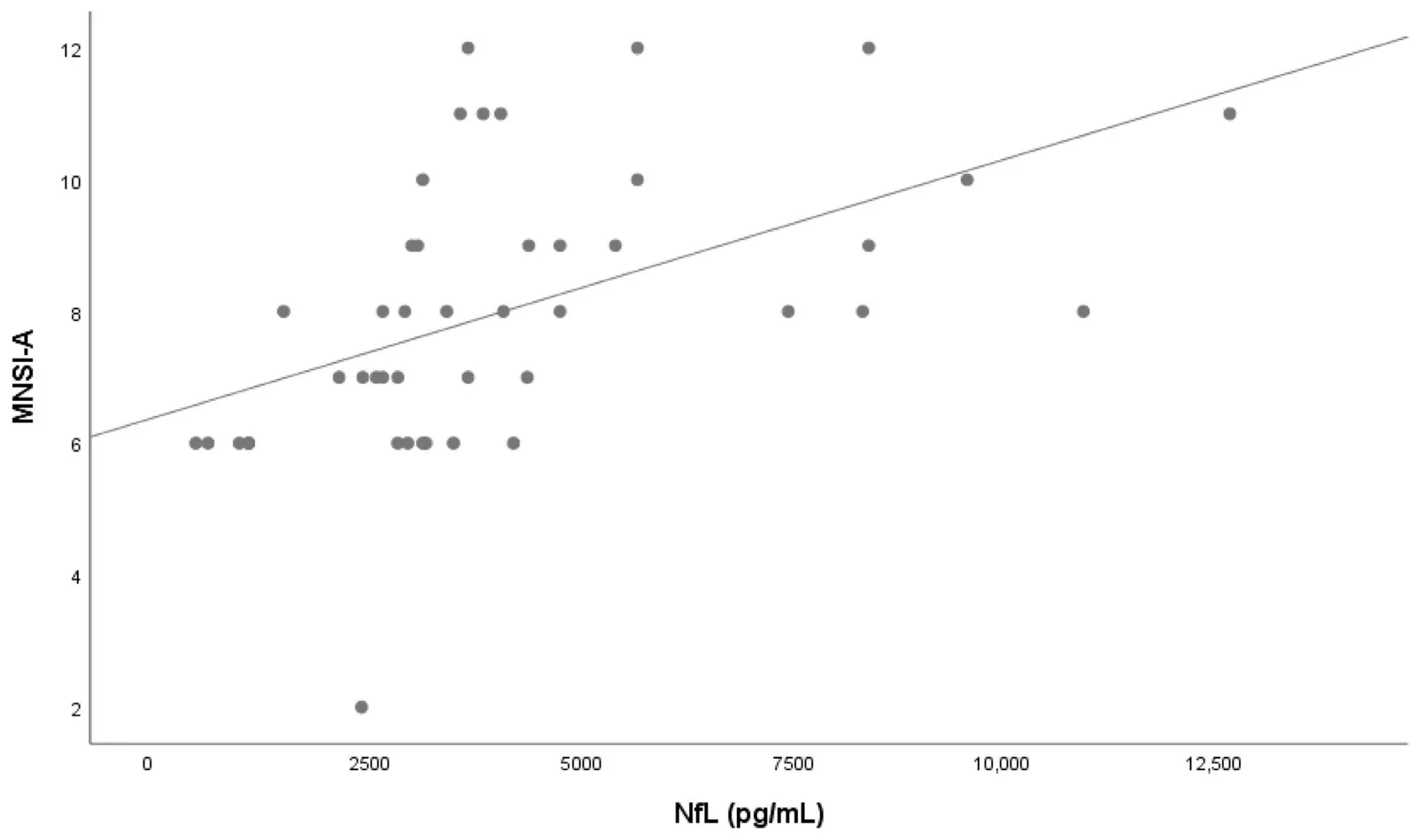
Association between serum NfL concentrations and MNSI-A scores in participants with DPN (Spearman ρ = 0.637; *p* < 0.001). Dots represent individual participant observations, and the line represents the fitted trend line.

In exploratory analyses restricted to participants with DPN, higher NfL was associated with greater neuropathy symptoms and examination burden, greater fear of falling, poorer balance performance, and fall history, with an additional inverse association with BMI. No clear association was observed with categorical electrophysiological severity (Table 6). Within the DPN subgroup, serum NfL showed a significant positive correlation with MNSI-A scores (ρ = 0.637; *p* < 0.001) (Figure 3).

## 4. Discussion

The present analytical cross-sectional findings indicate that circulating NfL concentrations were higher in participants with DPN and were associated with several measures of neuropathy and functional burden. However, comprehensive adjustment attenuated the association between NfL and DPN, and the confidence interval included the null value. These results support cautious interpretation of NfL as a potential complementary marker rather than definitive evidence of an independent or standalone diagnostic biomarker.

The higher NfL concentrations observed in participants with DPN are in agreement with accumulating evidence linking circulating NfL to diabetic peripheral nerve involvement. Maalmi et al. demonstrated relationships between serum NfL, diabetic sensorimotor polyneuropathy, and peripheral nerve dysfunction [7]. Määttä et al. likewise reported higher NfL concentrations in individuals with type 2 diabetes and DPN and identified relationships with abnormalities in nerve conduction [8]. Evidence from longitudinal observations further suggests that changes in NfL over time may be related to subsequent DPN risk [9]. More recently, Chen et al. found increased serum NfL in DPN and identified NfL as a factor independently related to the disorder [14]. Viewed alongside these reports, our results provide further evidence that elevated circulating NfL accompanies the neuroaxonal involvement associated with DPN [15].

The relationship between NfL and neuropathy burden was apparent for the MNSI measures. However, because MNSI findings contributed to the overall clinical assessment used in DPN classification, incorporation or circularity cannot be completely excluded. NfL–MNSI associations should therefore be interpreted as exploratory associations with neuropathy symptom and examination burden rather than independent validation of NfL diagnostic utility.

An important aspect of the study is the relationship between NfL and functional performance. Higher NfL concentrations were accompanied by lower BBS scores and higher FES-I scores, with similar patterns observed in the DPN subgroup. Loss of peripheral sensation, impaired proprioceptive input, and motor dysfunction in DPN can compromise postural control and thereby contribute to balance impairment and susceptibility to falling [3,4]. Previous investigations have documented impaired balance and increased fall risk in DPN [3,4], and the systematic review by Snopek Khan and Andersen further highlighted adverse effects of diabetic neuropathy on postural balance and daily functioning as well as its relationship with falls [16]. Our findings extend this clinical evidence by demonstrating that a circulating marker of neuroaxonal injury is related to both an objective measure of balance and a patient-reported measure of fear of falling. Whether serum NfL has value for anticipating subsequent functional deterioration, however, requires longitudinal investigation.

Higher NfL concentrations were associated with a greater number of retrospectively self-reported falls during the preceding 12 months. Because falls were assessed retrospectively, recall bias is possible, and this cross-sectional study cannot establish whether serum NfL predicts future falls.

Serum NfL concentrations did not differ significantly across electrophysiological DPN severity categories. This exploratory analysis was limited by small and unequal groups, particularly the severe group (*n* = 5), and the nonsignificant result should not be interpreted as evidence of no association.

Interpretation of circulating NfL also requires consideration of factors beyond peripheral neuropathy itself. Age and kidney function, in particular, may alter blood NfL concentrations. Ciardullo et al. reported higher serum NfL among individuals with diabetes and identified age, sex, and eGFR as independent correlates of circulating NfL [17]. Reduced eGFR has also been linked to higher NfL concentrations [17]. We attempted to limit this source of confounding by excluding participants with eGFR < 60 mL/min/1.73 m^2^ and incorporating eGFR into the fully adjusted analysis. Nonetheless, the influence of demographic, renal, and other biological factors complicates the establishment of a single NfL threshold applicable across different clinical populations.

BMI represents another factor requiring consideration. An inverse relationship between BMI and serum NfL was observed in our cohort and was stronger among participants with DPN. Manouchehrinia et al. similarly reported lower circulating NfL with increasing BMI and proposed that BMI and blood volume may influence interpretation of blood NfL measurements [18]. Our results are directionally consistent with this observation. However, those earlier data were obtained outside a DPN-specific population, and the current cross-sectional analysis cannot determine the mechanism or clinical significance of the inverse relationship between BMI and NfL in diabetic neuropathy.

The multivariable analyses illustrate the need for cautious interpretation of NfL as a DPN biomarker. In the comprehensively adjusted model, HbA1c remained associated with DPN, whereas the NfL effect estimate remained positive but statistical evidence was attenuated (aOR = 1.352; 95% CI, 0.991–1.844; *p* = 0.057). These findings should be considered alongside previous work evaluating NfL in combination with standard clinical variables for the prediction of diabetic polyneuropathy [19].

ROC analysis showed moderate discrimination (AUC = 0.716). Although median NfL was higher in DPN, the distributions showed substantial overlap. The exploratory threshold of ≥2.823 pg/mL was derived and evaluated in the same cohort and may therefore be optimistic; it should not be interpreted as a clinically validated diagnostic cut-off. Absolute NfL concentrations should also be interpreted in the context of the analytical platform and relevant biological and clinical factors.

### Strengths and Limitations

A distinctive feature of this study is the simultaneous evaluation of serum NfL in relation to several dimensions of DPN within the same cohort. Rather than examining only DPN status, we considered neuropathic symptoms and clinical findings, objective balance performance, fear of falling, and previous falls. Potential influences on circulating NfL, including glycemic control, BMI, and renal function, were also considered in the analyses.

Several limitations should be acknowledged. The analytical cross-sectional design prevents causal or prospective inference. Falls were retrospectively self-reported and are susceptible to recall bias. The single-center sample was relatively small, and the severe DPN subgroup included only five participants. Exclusion of suspected isolated small-fiber neuropathy without electrophysiological large-fiber involvement may have introduced spectrum bias and limits generalizability to isolated small-fiber neuropathy. Individual nerve-specific amplitudes, conduction velocities, distal latencies, and reference ranges were not available in a structured form for separate analysis. No formal correction for multiple comparisons was applied to exploratory correlations, increasing the possibility of type I error. Because MNSI contributed to the overall DPN assessment, incorporation bias may have strengthened NfL–MNSI associations. Vascular disease, other diabetes-related complications, medication use, physical activity, and other unmeasured factors were not comprehensively characterized for adjustment; residual confounding cannot be excluded.

In addition, circulating NfL can be affected by characteristics unrelated to DPN, including age, body composition, and renal function, and residual confounding cannot be excluded despite statistical adjustment. NfL was quantified using an ELISA-based assay; consequently, absolute concentrations and the threshold obtained in this study may not be directly interchangeable with values generated by other analytical platforms. Finally, the ≥2.823 pg/mL threshold was derived and evaluated within the same cohort and therefore represents a study-specific estimate rather than a clinically validated diagnostic cut-off. External validation in independent populations is required.

Prospective studies with larger samples, detailed nerve conduction measurements, and repeated NfL assessments would help clarify whether changes in NfL track DPN progression and whether they precede clinically important outcomes. In particular, longitudinal evaluation of balance deterioration and incident falls could determine whether the cross-sectional functional relationships identified here have prognostic relevance.

## 5. Conclusions

In this analytical cross-sectional study, serum NfL concentrations were higher in participants with T2DM and DPN and were associated with greater neuropathy symptoms and examination burden, poorer balance, greater fear of falling, and a history of falls. However, the association between NfL and DPN was attenuated after comprehensive adjustment, and the internally derived ROC threshold requires external validation. Serum NfL should therefore be considered a potential complementary marker rather than a standalone diagnostic biomarker.

## Figures and Tables

**Table 1:** Demographic, clinical, functional, and laboratory characteristics according to DPN status.

Variable	DPN (+) (*n* = 44)	DPN (−) (*n* = 44)	*p*-Value
Age (years), mean ± SD	60.27 ± 10.07	58.36 ± 9.33	0.359
Sex, female/male, n	26/18	38/6	0.008
BMI (kg/m^2^), mean ± SD	30.76 ± 4.86	31.85 ± 5.25	0.316
Diabetes duration (months), median (IQR)	120 (57–180)	72 (36–126)	0.143
HbA1c (%), median (IQR)	7.65 (6.75–9.30)	6.65 (6.40–7.33)	0.001
eGFR (mL/min/1.73 m^2^), median (IQR)	90.22 (83.73–95.13)	94.73 (86.48–100.98)	0.093
Serum NfL (pg/mL), median (IQR)	3.47 (2.68–4.77)	2.47 (1.48–3.07)	<0.001
Berg Balance Scale, median (IQR)	44 (42–45.25)	48 (47–51.25)	<0.001
FES-I, median (IQR)	34 (32–38.25)	23.5 (20–25.75)	<0.001
Number of falls, median (IQR)	1 (0–3)	0 (0–0.25)	<0.001

Note. Values are presented as mean ± standard deviation (SD), median (interquartile range [IQR]), or number, as appropriate. BMI, body mass index; DPN, diabetic polyneuropathy; eGFR, estimated glomerular filtration rate; FES-I, Falls Efficacy Scale–International; NfL, neurofilament light chain. For the primary between-group comparison of serum NfL, the median difference was 1.00 pg/mL (95% CI, 0.40–1.81), with a rank-biserial correlation of 0.43 (95% CI, 0.21–0.64).

**Table 2:** Correlation analysis between serum NfL levels and clinical, neuropathy-related, and functional assessment parameters.

Variable	Spearman ρ	Approximate 95% CI	*p*-Value
Age (years)	0.144	−0.067 to 0.343	0.179
BMI (kg/m^2^)	−0.312	−0.489 to −0.110	0.003
Diabetes duration (months)	0.100	−0.112 to 0.303	0.353
HbA1c (%)	0.307	0.104 to 0.485	0.004
eGFR (mL/min/1.73 m^2^)	−0.103	−0.306 to 0.109	0.339
MNSI-A	0.562	0.400 to 0.690	<0.001
MNSI-B	0.550	0.385 to 0.681	<0.001
BBS	−0.450	−0.603 to −0.266	<0.001
FES-I	0.431	0.244 to 0.587	<0.001
Number of falls	0.344	0.145 to 0.516	0.001

Note. NfL, neurofilament light chain; BMI, body mass index; eGFR, estimated glomerular filtration rate; MNSI, Michigan Neuropathy Screening Instrument; BBS, Berg Balance Scale; FES-I, Falls Efficacy Scale–International. Approximate 95% confidence intervals for Spearman correlation coefficients were calculated using Fisher’s z transformation. No formal multiplicity correction was applied to these exploratory correlation analyses.

**Table 3:** Fully adjusted logistic regression analysis of factors associated with diabetic polyneuropathy.

Variable	Adjusted OR	95% CI	*p*-Value
Age (years)	1.028	0.972–1.087	0.335
Sex (male)	3.257	0.861–12.316	0.082
BMI (kg/m^2^)	1.029	0.925–1.145	0.593
Diabetes duration (months)	1.001	0.994–1.008	0.792
HbA1c (%)	1.815	1.158–2.843	0.009
eGFR (mL/min/1.73 m^2^)	0.985	0.951–1.019	0.382
Serum NfL (pg/mL)	1.352	0.991–1.844	0.057

Note. DPN status was the dependent variable. The model simultaneously included age, sex, BMI, diabetes duration, HbA1c, eGFR, and serum NfL. Covariates were selected on the basis of clinical and biological relevance as potential confounders rather than univariable statistical significance. OR, odds ratio; CI, confidence interval; BMI, body mass index; eGFR, estimated glomerular filtration rate; NfL, neurofilament light chain. For serum NfL, the adjusted OR represents the change in the odds of DPN associated with a 1-pg/mL increase in NfL.

**Table 4:** ROC analysis of serum NfL for discrimination of diabetic polyneuropathy.

Variable	AUC (95% CI)	*p*-Value	Cut-Off (pg/mL)	Sensitivity (%)	Specificity (%)
Serum NfL	0.716 (0.607–0.825)	<0.001	≥2.823	72.7	65.9

NfL: neurofilament light chain; AUC: area under the receiver operating characteristic curve; CI: confidence interval. The threshold was derived internally using the Youden index and is exploratory.

**Table 5:** Exploratory comparison of demographic, clinical, and functional characteristics according to electrophysiological DPN severity.

Variable	Mild (*n* = 20)	Moderate (*n* = 19)	Severe (*n* = 5)	*p*-Value	Post Hoc
Duration of diabetes (months)	60.00 (40.50–120.00)	144.00 (96.00–192.00)	120.00 (61.00–240.00)	0.018	1 < 2
HbA1c (%)	6.80 (6.25–9.52)	8.10 (7.30–8.90)	9.30 (6.75–9.80)	0.058	
eGFR (mL/min/1.73 m^2^)	90.58 (85.04–95.37)	90.29 (82.77–101.82)	89.65 (67.55–93.76)	0.911	
MNSI-A	7.00 (6.00–8.00)	8.00 (7.00–10.00)	11.00 (8.00–12.00)	0.009	1 < 2 = 3
MNSI-B	2.50 (2.50–3.50)	3.00 (2.50–3.50)	4.50 (3.25–5.50)	0.332	
Serum NfL (pg/mL)	3.03 (2.42–4.35)	3.80 (2.85–5.68)	3.68 (2.87–6.91)	0.152	
BBS	45.00 (43.00–46.75)	43.00 (41.00–45.00)	40.00 (40.00–42.50)	0.014	1 > 2 = 3
FES-I	32.00 (30.00–34.75)	35.00 (32.00–40.00)	39.00 (36.50–40.00)	0.019	1 < 3
Number of falls	0.00 (0.00–1.00)	2.00 (1.00–3.00)	4.00 (3.50–4.50)	0.002	1 < 2 < 3

Note. Data are presented as median (IQR). Groups 1, 2, and 3 correspond to mild, moderate, and severe electrophysiological DPN, respectively. Post hoc comparisons are shown as reported in the source analysis. BBS, Berg Balance Scale; DPN, diabetic polyneuropathy; eGFR, estimated glomerular filtration rate; FES-I, Falls Efficacy Scale–International; MNSI, Michigan Neuropathy Screening Instrument; NfL, neurofilament light chain. Given the small and unequal subgroup sizes, particularly the severe group (*n* = 5), these analyses should be interpreted as exploratory.

**Table 6:** Correlations between serum NfL levels and clinical, electrophysiological, and functional parameters in participants with DPN.

Variable	Spearman ρ	Approximate 95% CI	*p*-Value
Age (years)	0.150	−0.154 to 0.428	0.331
BMI (kg/m^2^)	−0.519	−0.707 to −0.263	<0.001
Diabetes duration (months)	0.051	−0.250 to 0.343	0.741
HbA1c (%)	0.179	−0.125 to 0.452	0.244
eGFR (mL/min/1.73 m^2^)	0.069	−0.233 to 0.359	0.657
Electrophysiological severity	0.245	−0.056 to 0.505	0.109
MNSI-A	0.637	0.419 to 0.785	<0.001
MNSI-B	0.525	0.270 to 0.711	<0.001
BBS	−0.447	−0.657 to −0.173	0.002
FES-I	0.466	0.196 to 0.670	0.001
Number of falls	0.342	0.050 to 0.580	0.023

Note. NfL, neurofilament light chain; BMI, body mass index; eGFR, estimated glomerular filtration rate; MNSI, Michigan Neuropathy Screening Instrument; BBS, Berg Balance Scale; FES-I, Falls Efficacy Scale–International. Electrophysiological severity refers to the mild/moderate/severe classification reported in the original nerve conduction study reports. Approximate 95% confidence intervals for Spearman correlation coefficients were calculated using Fisher’s z transformation. No formal multiplicity correction was applied to these exploratory analyses.

## Data Availability

The datasets generated and/or analyzed during the current study are not publicly available but are available from the corresponding author upon reasonable request.
